# Quality-of-care audits and perinatal mortality in South Africa

**DOI:** 10.2471/BLT.14.144683

**Published:** 2015-03-31

**Authors:** Emma R Allanson, Robert C Pattinson

**Affiliations:** a School of Women’s and Infants’ Health, University of Western Australia, King Edward Memorial Hospital, 374 Bagot Road, Subiaco, WA 6008, Australia.; b South African Medical Research Council, Maternal and Infant Health Care Strategies Unit, Cape Town, South Africa.

## Abstract

**Problem:**

Suboptimal care contributes to perinatal mortality rates. Quality-of-care audits can be used to identify and change suboptimal care, but it is not known if such audits have reduced perinatal mortality in South Africa.

**Approach:**

We investigated perinatal mortality trends in health facilities that had completed at least five years of quality-of-care audits. In a subset of facilities that began audits from 2006, we analysed modifiable factors that may have contributed to perinatal deaths.

**Local setting:**

Since the 1990s, the perinatal problem identification programme has performed quality-of-care audits in South Africa to record perinatal deaths, identify modifiable factors and motivate change.

**Relevant changes:**

Five years of continuous audits were available for 163 facilities. Perinatal mortality rates decreased in 48 facilities (29%) and increased in 52 (32%). Among the subset of facilities that began audits in 2006, there was a decrease in perinatal mortality of 30% (16/54) but an increase in 35% (19/54). Facilities with increasing perinatal mortality were more likely to identify the following contributing factors: patient delay in seeking help when a baby was ill (odds ratio, OR: 4.67; 95% confidence interval, CI: 1.99–10.97); lack of use of antenatal steroids (OR: 9.57; 95% CI: 2.97–30.81); lack of nursing personnel (OR: 2.67; 95% CI: 1.34–5.33); fetal distress not detected antepartum when the fetus is monitored (OR: 2.92; 95% CI: 1.47–5.8) and poor progress in labour with incorrect interpretation of the partogram (OR: 2.77; 95% CI: 1.43–5.34).

**Lessons learnt:**

Quality-of-care audits were not shown to improve perinatal mortality in this study.

## Résumé

**Problème:**

Des soins sous-optimaux contribuent à des taux élevés de mortalité périnatale. Les contrôles de la qualité des soins peuvent permettre de déterminer si des soins sont sous-optimaux et de les modifier, mais il reste à savoir si ce type de contrôle a permis de réduire la mortalité périnatale en Afrique du Sud.

**Approche:**

Nous avons examiné les tendances en termes de mortalité périnatale dans des établissements de santé qui avaient réalisé des contrôles de la qualité des soins sur au moins cinq ans. Dans un sous-groupe d'établissements ayant commencé ces contrôles dès 2006, nous avons analysé les facteurs modifiables qui pourraient avoir eu un effet sur la mortalité périnatale.

**Environnement local:**

Depuis les années 1990, dans le cadre du programme d'identification des problèmes périnataux (PPIP), des contrôles de la qualité des soins ont été réalisés en Afrique du Sud afin d'enregistrer les décès périnataux, de déterminer les facteurs modifiables et de favoriser des changements.

**Changements significatifs:**

Des contrôles en continu sur cinq ans avaient été réalisés dans 163 établissements. Le taux de mortalité périnatale avait diminué dans 48 établissements (29 %) et augmenté dans 52 (32 %). Concernant le sous-groupe des établissements qui avaient commencé le contrôle en 2006, on a observé une diminution de la mortalité périnatale dans 30 % d'entre eux (16/54) mais une augmentation dans 35 % de ces établissements (19/54). Dans les établissements qui affichaient une augmentation du taux de mortalité périnatale, les facteurs suivants étaient plus fréquemment identifiés : consultation tardive des patients lorsqu'un enfant était malade (rapport des cotes, RC : 4,67 ; intervalle de confiance de 95 %, IC : 1,99–10,97) ; non-administration prénatale de stéroïdes (RC : 9,57 ; IC de 95 % : 2,97–30,81) ; manque de personnel infirmier (RC : 2,67 ; IC de 95 % : 1,34–5,33) ; souffrance fœtale non détectée ante partum lors de la surveillance du fœtus (RC : 2,92 ; IC de 95 % : 1,47–5,8) et mauvaise progression du travail, avec une interprétation incorrecte du partogramme (RC : 2,77 ; IC de 95 % : 1,43-5,34).

**Leçons tirées:**

Cette étude n'a pas montré que le contrôle de la qualité des soins permettait de réduire la mortalité périnatale.

## Resumen

**Situación:**

El cuidado por debajo del nivel óptimo contribuye a las tasas de mortalidad perinatal. Las verificaciones de la calidad de la asistencia se puede utilizar para identificar y cambiar el cuidado por debajo del nivel óptimo, pero no se sabe si tales verificaciones han reducido la mortalidad perinatal en Sudáfrica.

**Enfoque:**

Se investigaron las tendencias de mortalidad perinatal en centros de salud que habían completado por lo menos cinco años de verificaciones de la calidad de la asistencia. En un subgrupo de centros que empezaron las verificaciones en 2006, se analizaron los factores modificables que podrían haber contribuido a las muertes perinatales.

**Marco regional:**

Desde la década de 1990, el programa de identificación del problema perinatal ha realizado verificaciones de la calidad de la asistencia en Sudáfrica para registrar las muertes perinatales, identificar los factores modificables y estimular el cambio.

**Cambios importantes:**

Cinco años de verificaciones continuas estuvieron disponibles para 163 centros. Las tasas de mortalidad perinatal disminuyeron en 48 centros (28%) y aumentaron en 52 (32%). En el subgrupo de centros que empezó la verificación en 2006, hubo una disminución en la mortalidad perinatal del 30% (16/54), pero un aumento del 35% (19/54). Los centros con una mortalidad perinatal en aumento tenían una mayor probabilidad de identificar los siguientes factores: retraso de los pacientes en la búsqueda de ayuda cuando un niño enfermaba (cociente de posibilidades, CP: 4,67; intervalo de confianza, IC, del 95%: 1,99–10,97); falta de uso de asteroides prenatales (CP: 9,57 (IC del 95%: 2,97–30,81); falta de personal de enfermería (CP: 2,67 (IC del 95%: 1,34–5,33); septicemia neonatal no identificada antes del parto durante el control del feto (CP: 2,92 (IC del 95%: 1,47–5,8) y escasos progresos en el parto con una interpretación incorrecta del partograma (CP: 2,77 (IC del 95%: 1,43–5,34).

**Lecciones aprendidas:**

Las verificaciones de la calidad de la asistencia no ha mostrado mejoras en la mortalidad perinatal en este estudio.

## ملخص

**المشكلة:**

تسهم الرعاية التي تكون دون المستوى في زيادة معدلات الوفيات المحيطة بالولادة **. ** يمكن استخدام مراجعات جودة الرعاية لتحديد وتغيير الرعاية التي تكون دون المستوى، ولكن من غير المعروف ما إذا كانت هذه المراجعات قد قامت بالحد من الوفيات المحيطة بالولادة في جنوب أفريقيا أم لا.

**الأسلوب:**

قمنا باستقصاء نزعات الوفيات المحيطة بالولادة في المرافق الصحية التي أكملت خمس سنوات على الأقل من تنفيذ مراجعات جودة الرعاية. في مجموعة ثانوية من المرافق التي بدأت في تنفيذ عمليات المراجعة منذ عام 2006، قمنا بتحليل العوامل القابلة للتعديل التي ربما تكون قد ساهمت في الوفيات المحيطة بالولادة.

**المواقع المحلية:**

منذ التسعينيات، قام برنامج تحديد المشكلة المحيطة بالولادة بتنفيذ عمليات مراجعة جودة الرعاية في جنوب أفريقيا لتسجيل الوفيات المحيطة بالولادة، وتحديد العوامل القابلة للتعديل، وتحفيز التغيير.

**التغيّرات ذات الصلة:**

توفرت خمس سنوات من المراجعات المستمرة لعدد 163 مِرفقًا. انخفضت معدلات الوفيات المحيطة بالولادة في 48 مرفقًا (‏‏29٪) وارتفعت المعدلات في 52 مرفقًا (‏‏32٪). من بين المجموعة الثانوية من المرافق التي بدأت في تنفيذ المراجعات في عام 2006، كان هناك انخفاض في معدل الوفيات المحيطة بالولادة بنسبة بلغت 30٪ (16/54)، ولكن كان هناك زيادة بنسبة تبلغ 35‏٪ (19/54). كانت المرافق التي تعاني من معدلات متزايدة للوفيات المحيطة بالولادة، أكثر أرجحية لتحديد العوامل المساهمة التالية: تأخر المريض في طلب المساعدة عندما كان الطفل مريضًا (نسبة الاحتمال: 4.67؛ بنسبة أرجحية مقدارها ‏95‏%: 1.99 إلى 10.97)؛ الافتقار لاستخدام الستيرويدات في الفترة السابقة للولادة (نسبة الاحتمال: 9.57؛ بنسبة أرجحية مقدارها ‏95%: 2.97 إلى 30.81)؛ قلة أعداد العاملين في مجال التمريض (نسبة الاحتمال: 2.67؛ بنسبة أرجحية مقدارها 95%: 1.34 إلى 5.33)؛ لم يتم اكتشاف المشكلات الجنينية في الفترة السابقة للولادة عندما يتم رصد الجنين (نسبة الاحتمال: 2.92؛ بنسبة أرجحية مقدارها ‏95%: 1.47 إلى 5.8) والتقدم الضعيف في المخاض، إلى جانب التفسير الخاطئ لمخطط الولادة (نسبة الاحتمال: 2.77؛ بنسبة أرجحية مقدارها ‏95%: 1.43 إلى 5.34).

**الدروس المستفادة:**

لم يتضح تأثير لمراجعات جودة العناية في تحسين معدلات الوفيات المحيطة بالولادة في هذه الدراسة.

## 摘要

**问题:**

医疗情况不理想是导致围产儿死亡率上升的因素。医疗质量审核可用于确定并改变不理想的医疗情况，但是目前尚不清楚该种审核是否能降低南非境内的围产儿死亡率。

**方法:**

我们在已经完成医疗质量审核至少长达五年的医疗机构中调查了围产儿的死亡率变化趋势。针对从 2006 年开始审核的一部分机构，我们分析了造成围产儿死亡的可变因素。

**当地状况:**

自二十世纪九十年代以来，围产儿问题确定计划已在南非开展医疗质量审核，记录围产儿死亡情况，确定可变因素并促进改变。

**相关变化:**

五年来持续为 163 家机构提供审核。48 家机构 (29%) 的围产儿死亡率下降，52 家机构 (32%) 的围产儿死亡率上升。在从 2006 年开始审核的一些机构中，30% (16/54) 的机构出现围产儿死亡率下降，但是 35% (19/54) 的机构却出现围产儿死亡率上升。围产儿死亡率上升的机构更有可能与以下确定的诱发因素有关：婴儿生病时，患者就医延迟（比值比，OR：4.67；95% 置信区间，CI：1.99–10.97）；没有使用产前类固醇 (OR:9.57；95% CI：2.97–30.81)；缺乏护理人员 (OR:2.67；95% CI：1.34–5.33)；没有在产前对胎儿进行监护时检测出胎儿困厄的情况 (OR:2.92; 95% CI:1.47–5.8)；以及产程进展缓慢，工作人员无法正确判读产程图 (OR:2.77; 95% CI: 1.43–5.34)。

**经验教训:**

医疗质量审核尚未在本研究中表明围产儿死亡率情况得到改善的结果。

## Резюме

**Проблема:**

Недостаточный уровень медицинской помощи способствует росту показателей перинатальной смертности. Аудит качества медицинской помощи можно использовать для выявления недостаточного медицинского ухода и возможного изменения сложившейся ситуации. Тем не менее эффективность такого аудита для уменьшения уровня перинатальной смертности в Южной Африке неизвестна.

**Подход:**

Были исследованы тенденции в отношении перинатальной смертности в тех учреждениях здравоохранения, которые проводили аудит качества медицинской помощи по меньшей мере в течение пяти лет. В подгруппе учреждений, начавших аудит с 2006 года, были проанализированы модифицируемые факторы, которые могли вносить вклад в показатели перинатальной смертности.

**Местные условия:**

В Южной Африке с 90-х годов ХХ века проводится аудит качества медицинской помощи по программе идентификации перинатальных проблем с целью учета уровня перинатальной смертности, выявления модифицируемых факторов и стимулирования изменений.

**Осуществленные перемены:**

Были получены результаты непрерывного пятилетнего аудита из 163 учреждений. Показатели перинатальной смертности снизились в 48 учреждениях (29 %) и выросли в 52 (32 %). Среди подгруппы учреждений, которые приступили к аудиту в 2006 году, снижение перинатальной смертности выявлено в 30 % случаев (16 из 54 учреждений), однако в 35 % (19 из 54 учреждений) выявлен рост. В тех учреждениях, где отмечался рост перинатальной смертности, с большей вероятностью выявлялись следующие факторы: задержка с обращением за помощью со стороны пациентов, когда ребенок заболел (отношение шансов, ОШ: 4,67; 95 % доверительный интервал, ДИ: 1,99–10,97); недостаточное использование стероидов в антенатальном периоде (ОШ: 9,57; 95 % ДИ: 2,97–30,81); нехватка младшего медицинского персонала (ОШ: 2,67; 95 % ДИ: 1,34–5,33); невыявленное при дородовом мониторинге угнетенное состояние плода (ОШ: 2,92; 95 % ДИ: 1,47–5,8); плохая динамика родов с неверной интерпретацией партограммы (ОШ: 2,77; 95 % ДИ: 1,43–5,34).

**Выводы:**

По результатам данного исследования аудит качества медицинской помощи не привел к улучшению показателей перинатальной смертности.

## Introduction

Perinatal mortality in South Africa remains high, with 33.4 deaths per 1000 live births in 2013.^1^^–^^3^ Quality-of-care audits have been shown in non-randomized trials to reduce perinatal mortality by up to 30%.^4^^–^^6^ The audit includes classifying avoidable deaths, changing service delivery and addressing health system problems.

The South African Medical Research Council introduced the Perinatal Problem Identification Program in the 1990s to capture perinatal mortality, identify modifiable factors and motivate change.^7^ This programme is a part of a quality-of-care audit cycle and until 2012 participation was voluntary. The programme is used at all levels of care, and captured 94% of hospitals (238/252) and 73% of births (1 330 869/1 820 664).^8^ We wanted to determine how perinatal mortality rates had changed in health-care facilities participating in the perinatal problem identification programme.

## Approach

We used data from the programme to explore the impact of onsite quality-of-care audits on the perinatal mortality rate, which is defined as fetal and early neonatal deaths (0–7 days) per 1000 births. We analysed perinatal mortality rates of babies weighing more than 1000 g from 163 facilities with at least five years of continuous audits between 1990 and 2013. There were 3 406 347 births and 85 728 deaths from 29 community health centres, 105 district hospitals, 4 national central hospitals, 22 regional hospitals and three provincial tertiary hospitals.

Data were smoothed using 12 month moving averages; trends in mortality were analysed using Epi Info version 7 (Centers for Disease Control and Prevention, Atlanta, United States of America). SPSS version 22 (IBM Corp., Armonk, USA) was used for all other analyses. For each site, we tested for temporal trends in perinatal mortality rates using the extended Mantel-Haenszel *M^2^* statistic with one degree of freedom. The trend was assumed to be monotonic (i.e. continuously increasing or decreasing, compared to the initial value of the perinatal mortality rate). A *P-*value of less than 0.05 was considered significant.

Next, we tested the effect of the programme on a subgroup of 54 facilities that began auditing from 2006 onwards. We analysed two of the specific indicators of quality-of-care audits: the identification of modifiable factors in a death and the final obstetric cause of death. We compared facilities with increasing mortality and facilities with decreasing mortality.

The programme defines 69 modifiable factors which are an incident related to the actions of the mother or health-care personnel, or the health-care system, which may have altered the outcome of the specific case had it been managed differently.^9^ Clinical staff identify potentially modifiable factors in the immediate period after the death. We estimated the crude odds ratios (OR) for a modifiable factor being implicated in a death in facilities with increasing mortality compared with facilities with decreasing mortality. To account for multiple testing (since more than one modifiable factor may be identified per death), a *P-*value of less than 0.01 was considered significant.

We calculated the average number of modifiable factors per death and the rates of obstetric causes of death (per 1000 total deaths) in the first and fifth year of audit. Changes in these values over time were assessed using a *t*-test for independent samples.

The Perinatal Problem Identification Program has ethical approval from the University of Pretoria. Data were collected with permission from the South African Department of Health. This secondary analysis was approved by the technical task team of the South African Medical Research Council.

## Relevant changes

Of the 163 facilities, 29% (48) had a decreasing perinatal mortality rate, 32% (52) had an increasing rate and 39% (63) had no significant change. Included in these facilities were 29 community health centres (11 increasing, five decreasing and 13 no change), 105 district hospitals (32 increasing, 37 decreasing and 36 no change), 22 regional hospitals (seven increasing, five decreasing and 10 no change), four national central hospitals (one increasing, one decreasing and two no change) and three provincial tertiary hospitals (one increasing, two no change).

**Fig. 1:**
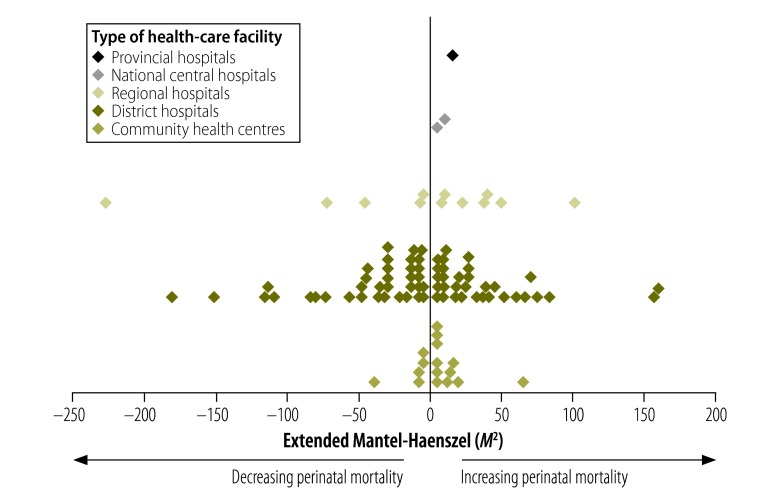
Trends in perinatal mortality for the first five years of quality-of-care audits in health-care facilities, South Africa

Note: Each diamond represents one health-care facility.

Fig. 1 shows the trend in perinatal mortality rates for facilities with a significant increase or decrease in mortality. One district hospital reduced its mortality rate from 100 deaths per 1000 live births at the beginning of the audits to 12 deaths per 1000 live births at the end of five years (smoothed data). As the site had only 2438 births (0.07% of total births) and 130 deaths (0.15% of total deaths) over the whole period, we did not remove these cases from subsequent analyses, however this site is omitted from Fig. 1.

In the 54 facilities that began auditing after 2006, 19 facilities (35%) had increasing mortality and 16 facilities (30%) had decreasing mortality. Facilities with increasing mortality were more likely to identify the following modifiable factors: patient delay in seeking help when a baby was ill (OR: 4.67; 95% confidence interval, CI: 1.99–10.97); lack of use of antenatal steroids (OR: 9.57; 95% CI: 2.97–30.81); lack of nursing personnel (OR: 2.67; 95% CI: 1.34–5.33); fetal distress not detected antepartum when the fetus is monitored (OR: 2.92; 95% CI: 1.47–5.8) and poor progress in labour with incorrect interpretation of the partogram (OR: 2.77; 95%: CI: 1.43–5.34). These same facilities were also significantly less likely to identify alcohol abuse, women who attended antenatal care late in pregnancy, inappropriate response to antepartum haemorrhage, inappropriate response to poor fetal movements, inappropriate response to rupture of membranes, infrequent visits to antenatal clinic, smoking, inadequate facilities in the nursery, inadequate resuscitation equipment, lack of transport, fetal distress not detected intrapartum when the fetus is not monitored, inadequate monitoring of the neonate, inadequate neonatal management plan and no response to positive syphilis serology test.

Facilities with increasing mortality identified 1.28 modifiable factors per death in their first year of audit and 1.49 factors in their fifth year (*P* = 0.431). Facilities with decreasing mortality identified 1.53 modifiable factors per death in the first year and 1.66 in the fifth year (*P* = 0.73). The rate of obstetric cause of death in the first year of audit was not significantly different between the two groups. In the fifth year of audit, facilities with decreasing mortality had significantly lower rates of spontaneous preterm labour and unexplained intrauterine death (Table 1).

**Table 1:** Obstetric causes of death in quality-of-care audits for health-care facilities with increasing and decreasing perinatal mortality, South Africa

Cause	Perinatal mortality per 1000 births						
	First year audit				Fifth year audit		
	Facilities with decreasing mortality	Facilities with increasing mortality	*P*		Facilities with decreasing mortality	Facilities with increasing mortality	*P*
Spontaneous preterm labour	5.0	3.0	0.150		3.0	5.8	0.038
Unexplained intrauterine death	5.5	4.1	0.487		3.3	6.9	0.011
Intrapartum asphyxia	3.1	3.5	0.713		3.1	5.8	0.07
Hypertension	3.3	0.9	0.090		1.1	2.1	0.106
Infections	1.4	1.6	0.785		0.4	0.8	0.250
Intrauterine growth restriction	0.3	0.1	0.311		0.1	0.1	0.582
Antepartum haemorrhage	1.2	0.8	0.535		1.1	1.8	0.210
Maternal disease	1.2	0.1	0.076		0.4	0.8	0.474
Fetal abnormality	0.7	0.2	0.209		0.9	0.6	0.550

## Lessons learnt

Audits are critical to the identification of potential problems; focused audits within a wider system can identify contextually specific service deficiencies and provide the impetus for change.^10^^–^^12^ The variation in mortality rates in the facilities with five years of continuous quality-of-care audits suggests that this process does not necessarily reduce mortality. Facilities with increasing perinatal mortality identified some modifiable factors which should be easily remediable once identified (e.g. using antenatal corticosteroids).

That the facilities with increasing mortality rates were less likely to identify several of the modifiable factors is difficult to explain. There are no obvious differences between the groups in terms of level of health care, numbers of births and the obstetric causes of death at the beginning of the audits. There were three community health centres with 39 151 births in sites with increasing mortality and community health centres with 11 168 births in sites with decreasing mortality (*P* = 0.137) and 16 district hospitals with 112 754 births in sites with increasing mortality and 12 district hospitals with 90 747 births in sites with decreasing mortality (*P* = 0.837).

We know from qualitative research that there are factors that make audits successful – team drivers, institutional review, feedback and communication within the system.^13^ We hypothesize that it is the quality of the process (the detailed death review and the response to modifiable factors) that is the vital component that changes perinatal mortality. This is supported by the significant reduction in the unexplained stillbirth category indicating a more thorough search for the cause of death.

The study has some limitations. Data were retrospective and so it was not possible to assess data accuracy or completeness of the review of perinatal deaths at each site. We did not adjust for temporal trends in maternal risk factors affecting perinatal mortality. Therefore, we cannot exclude the possibility that the observed changes in mortality were unrelated to clinical management.

In conclusion, we were unable to demonstrate an effect of quality-of-care audits on perinatal mortality. Further investigation of site response to audits and the effectiveness of mortality review needs to be undertaken to identify how best to use this tool, particularly in low- and middle-income settings with high perinatal mortality (Box 1).

Box 1 **Summary of main lessons learnt**

- We were unable to demonstrate an effect of quality-of-care audits on perinatal mortality.
- Facility-specific response to the audit process, including the response to identified modifiable factors, may be the critical step in reducing perinatal mortality.
- Further investigation is needed on how best to optimize quality-of-care audits as a tool in a low- and middle-income setting for reducing perinatal mortality.
